# Synthesis and crystal structure of *tert*-butyl 1-(2-iodo­benzo­yl)cyclo­pent-3-ene-1-carboxyl­ate

**DOI:** 10.1107/S2056989019011514

**Published:** 2019-08-30

**Authors:** Dejing Yin

**Affiliations:** a School of Biotechnology, Jiangnan University, Lihu Avenue 1800, Wuxi in Jiangsu Province, People’s Republic of China

**Keywords:** crystal structure, substrate, cyclo­pentene ring, hydrogen bonding

## Abstract

1-(2-Iodo­benzo­yl)-cyclo­pent-3-ene-1-carboxyl­ates are novel substrates to construct bi­cyclo­[3.2.1]octa­nes with anti­bacterial and anti­thrombotic activities. In this context, *tert*-butyl 1-(2-iodo­benzo­yl)-cyclo­pent-3-ene-1-carboxyl­ate, C_17_H_19_IO_3_, was synthesized and structurally characterized. The 2-iodo­benzoyl group is attached to the tertiary C atom of the cyclo­pent-3-ene ring. The dihedral angle between the benzene ring and the mean plane of the envelope-type cyclo­pent-3-ene ring is 26.0 (3)°. In the crystal, pairs of C-H⋯O hydrogen bonds link the mol­ecules to form inversion dimers.

## Abstract 2

The cyclo­pent-3-ene ring has an envelope conformation with the tertiary C atom as the flap atom.

## Chemical context   

1-(2-Iodo­benzo­yl)cyclo­pent-3-ene-1-carboxyl­ates were recently employed as novel substrates to construct bi­cyclo­[3.2.1]octa­nes that are widely found in natural products and bioactive mol­ecules with anti­bacterial and anti­thrombotic activities (Yuan *et al.*, 2019 ▸). Although the authors carried out some control experiments to reveal the reaction mechanism, crystal structures of the substrates have not been reported yet. Moreover, 1-(2-iodo­benzo­yl)cyclo­pent-3-ene is crucial to the reductive Heck reaction and thus may provide more direct information on this reaction mechanism if more detailed structural data are available. Herein, the synthesis and crystal structure of *tert*-butyl 1-(2-iodo­benzo­yl)cyclo­pent-3-ene-1-carboxyl­ate are reported.
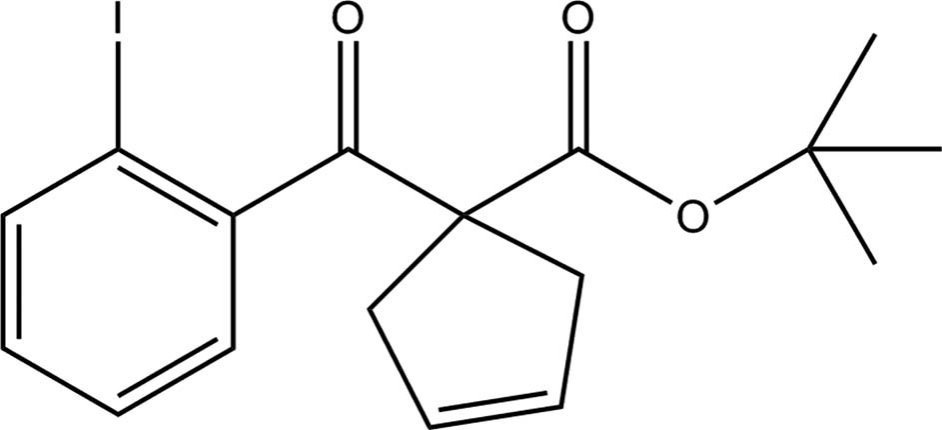


## Structural commentary   

**Figure 1:**
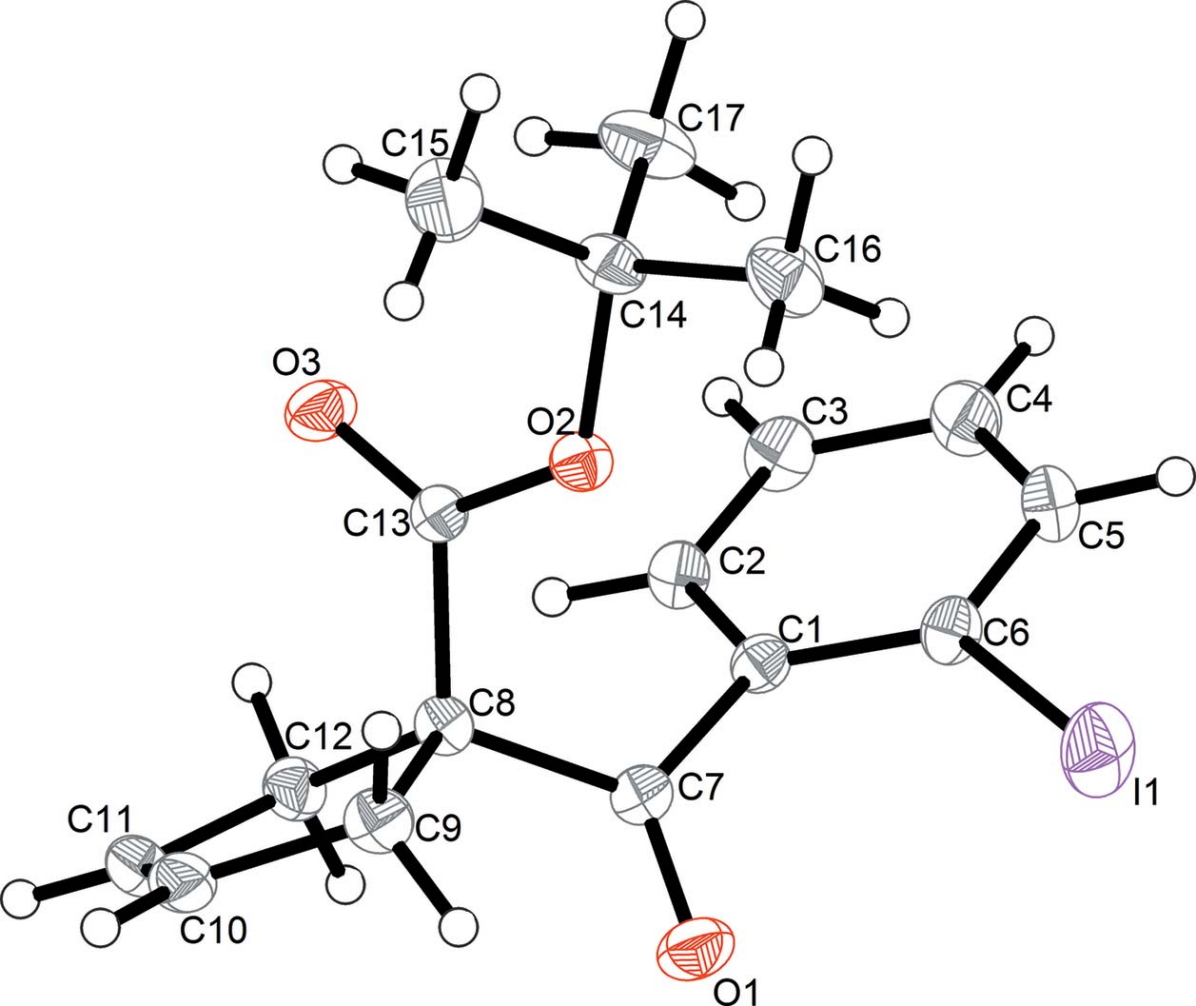
The mol­ecular structure of title compound, showing the atom-labelling scheme. Displacement ellipsoids are drawn at the 20% probability level.

The mol­ecular structure of the title compound is shown in Fig. 1 ▸. The 2-iodo­benzoyl group is attached to the tertiary C atom (C8) of the cyclo­pent-3-ene ring, with the *tert*-butyl carboxyl­ate group as the other substituent. The five-membered C8–C12 ring adopts an envelope conformation, with atom C8 as the flap, and with puckering parameters (Cremer & Pople, 1975 ▸) *Q* = 0.1526 Å and φ = 0.5354°, and pseudo-rotation parameters (Rao *et al.*, 1981 ▸) *P* = 162.5 (1)° and τ(M) = 15.2 (3)°. The deviation of C8 from the mean plane defined by atoms C9–C12 is 0.097 (4) Å. The dihedral angle between the benzene ring and the alkene plane (C9–C12) of the cyclo­pent-3-ene ring is 26.51 (19)°.

## Supra­molecular features   

**Figure 2:**
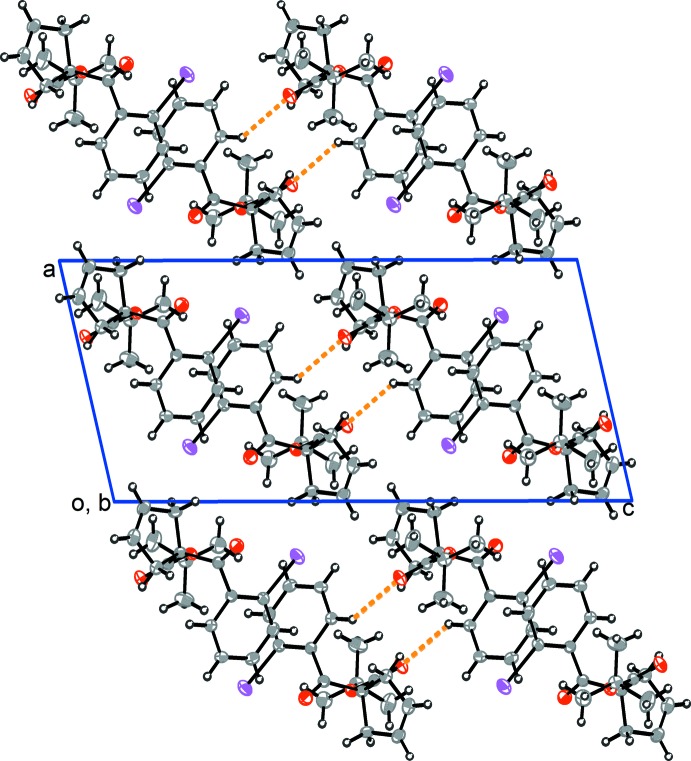
A view along [010] of the crystal packing of the title compound. The inter­molecular C—H⋯O hydrogen bonds are shown as orange dashed lines (Table 1 ▸).

In the crystal, mol­ecules are linked by a pair of C—H⋯O hydrogen bonds forming inversion dimers (Table 1 ▸ and Fig. 2 ▸). They stack up the *b* axis and form layers parallel to the *bc* plane. There are no other significant inter­molecular inter­actions present in the crystal.

## Database survey   

A search of the Cambridge Structural Database (CSD, Version 5.39, update of August 2018; Groom *et al.*, 2016 ▸) for entities containing (1-methyl­cyclo­pent-3-en-1-yl)(phen­yl)methanone yielded 27 hits. Only two of these compounds involve no other substituents at the cyclo­pent-3-ene ring as in the title compound, *viz*. methyl 4-[(1-methyl­cyclo­pent-3-en-1-yl)carbon­yl]benzoate in the space group *Pnma* (CSD refcode CIQHAM; Yang *et al.*, 2007 ▸), and 4-benzoyl-4-(meth­oxy­carbon­yl)cyclo­pentene in the space group *P*2_1_/*c*, with four independent mol­ecules in the asymmetric unit (CSD refcode KOGSIJ; Jiang *et al.*, 2008 ▸). In the structures of these two compounds, the folding angles of the cyclo­pent-3-ene ring are 17.00 (13) and 11.91 (12)°, respectively, while in the title compound it is 15.0 (3)°. The benzene ring in each structure is inclined to the alkene plane of the cyclo­pent-3-ene ring by 90.00 (8) and 61.40 (6)°, respectively, while the corresponding dihedral angle in the title compound is 26.51 (19)°. Apparently, different kinds of inter­molecular C—H⋯O hydrogen bonds and the presence or not of weak π–π contacts in the three structures lead to different mol­ecular packing and dihedral angles between the benzene ring and the cyclo­pent-3-ene ring.

## Synthesis and crystallization   

The title compound was prepared according to a general literature protocol (Yuan *et al.*, 2019 ▸). ^1^H NMR (300 MHz, CDCl_3_): δ 8.0 (*dd*, *J* = 7.9, 1.2 Hz, 1H), 7.4 (*dd*, *J* = 7.8, 1.8 Hz, 1H), 7.3 (*td*, *J* = 7.5, 1.2 Hz, 1H), 7.1 (*td*, *J* = 7.8, 1.8 Hz, 1H), 5.6 (*s*, 2H), 3.1 (*s*, 4H), 1.2 (*s*, 9H). HRMS (ESI) calcd for [C_17_H_19_IO_3_^+^Na]^+^ 421.0271, found 421.0272. Crystallization from a 5:1 mixture (*v*/*v*) of di­chloro­methane and *n*-hexane by slow evaporation at room temperature for about 7 d gave block-shaped crystals of the title compound.

## Refinement   

Crystal data, data collection and structure refinement details are summarized in Table 2 ▸. H atoms attached to C atoms were included in calculated positions and refined using a riding model: C—H = 0.93-0.97 Å with *U*_iso_(H) = 1.5*U*_eq_(C-meth­yl) and 1.2*U*_eq_(C) for other H atoms.

## Supplementary Material

Crystal structure: contains datablock(s) global, I. DOI: 10.1107/S2056989019011514/wm5513sup1.cif

Supporting information file. DOI: 10.1107/S2056989019011514/wm5513Isup2.cml

Structure factors: contains datablock(s) 20180530A_0m_a. DOI: 10.1107/S2056989019011514/wm5513Isup3.hkl

CCDC reference: 1899475

Additional supporting information:  crystallographic information; 3D view; checkCIF report

## Figures and Tables

**Table 1:** Hydrogen-bond geometry (Å, °)

*D*—H⋯*A*	*D*—H	H⋯*A*	*D*⋯*A*	*D*—H⋯*A*
C2—H2⋯O3^i^	0.93	2.48	3.219 (5)	136

Symmetry code: (i) 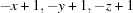.

**Table 2:** Experimental details

Crystal data	
Chemical formula	C_17_H_19_IO_3_
*M*_r_	398.22
Crystal system, space group	Monoclinic, *P*2_1_/*c*
Temperature (K)	299
*a*, *b*, *c* (Å)	9.4977 (2), 9.3635 (2), 19.8978 (4)
β (°)	102.752 (1)
*V* (Å^3^)	1725.90 (6)
*Z*	4
Radiation type	Cu *K*α
μ (mm^−1^)	14.64
Crystal size (mm)	0.3 × 0.2 × 0.1

Data collection	
Diffractometer	Bruker APEXII CCD
Absorption correction	Multi-scan (*SADABS*; Bruker, 2014 ▸)
*T*_min_, *T*_max_	0.262, 0.753
No. of measured, independent and observed [*I* > 2σ(*I*)] reflections	16056, 3278, 2612
*R*_int_	0.066
(sin θ/λ)_max_ (Å^−1^)	0.610

Refinement	
*R*[*F* ^2^ > 2σ(*F* ^2^)], *wR*(*F* ^2^), *S*	0.051, 0.131, 1.06
No. of reflections	3278
No. of parameters	193
H-atom treatment	H-atom parameters constrained
Δρ_max_, Δρ_min_ (e Å^−3^)	0.61, −1.22

Computer programs: *APEX3* and *SAINT* (Bruker, 2014 ▸), *SHELXT2014* (Sheldrick, 2015*a* ▸), *SHELXL2014* (Sheldrick, 2015*b* ▸), *DIAMOND* (Brandenburg, 2006 ▸) and *PLATON* (Spek, 2009 ▸) and *publCIF* (Westrip, 2010 ▸).

## supplementary crystallographic information

### Crystal data

**Table:**

C_17_H_19_IO_3_	*F*(000) = 792
*M**_r_* = 398.22	*D*_x_ = 1.533 Mg m^−^^3^
Monoclinic, *P*2_1_/*c*	Cu *K*α radiation, λ = 1.54178 Å
*a* = 9.4977 (2) Å	Cell parameters from 5961 reflections
*b* = 9.3635 (2) Å	θ = 4.6–69.9°
*c* = 19.8978 (4) Å	µ = 14.64 mm^−^^1^
β = 102.752 (1)°	*T* = 299 K
*V* = 1725.90 (6) Å^3^	Block, colourless
*Z* = 4	0.3 × 0.2 × 0.1 mm

### Data collection

**Table:**

Bruker APEXII CCD diffractometer	2612 reflections with *I* > 2σ(*I*)
φ and ω scans	*R*_int_ = 0.066
Absorption correction: multi-scan (SADABS; Bruker, 2014)	θ_max_ = 70.1°, θ_min_ = 4.6°
*T*_min_ = 0.262, *T*_max_ = 0.753	*h* = −10→11
16056 measured reflections	*k* = −11→11
3278 independent reflections	*l* = −24→22

### Refinement

**Table:**

Refinement on *F*^2^	Primary atom site location: dual
Least-squares matrix: full	Secondary atom site location: difference Fourier map
*R*[*F*^2^ > 2σ(*F*^2^)] = 0.051	Hydrogen site location: inferred from neighbouring sites
*wR*(*F*^2^) = 0.131	H-atom parameters constrained
*S* = 1.06	*w* = 1/[σ^2^(*F*_o_^2^) + (0.0548*P*)^2^ + 2.0895*P*] where *P* = (*F*_o_^2^ + 2*F*_c_^2^)/3
3278 reflections	(Δ/σ)_max_ < 0.001
193 parameters	Δρ_max_ = 0.61 e Å^−^^3^
0 restraints	Δρ_min_ = −1.22 e Å^−^^3^

### Special details

**Table:**

Geometry. All esds (except the esd in the dihedral angle between two l.s. planes) are estimated using the full covariance matrix. The cell esds are taken into account individually in the estimation of esds in distances, angles and torsion angles; correlations between esds in cell parameters are only used when they are defined by crystal symmetry. An approximate (isotropic) treatment of cell esds is used for estimating esds involving l.s. planes.

### Fractional atomic coordinates and isotropic or equivalent isotropic displacement parameters (Å^2^)

**Table:**

	*x*	*y*	*z*	*U*_iso_*/*U*_eq_	
I1	0.22879 (4)	0.41049 (5)	0.17053 (2)	0.0858 (2)	
O1	0.1852 (4)	0.6510 (4)	0.28314 (19)	0.0681 (9)	
C1	0.3928 (4)	0.5060 (4)	0.3155 (2)	0.0425 (8)	
O2	0.2177 (3)	0.3021 (3)	0.37481 (14)	0.0444 (6)	
C2	0.5151 (4)	0.5150 (5)	0.3679 (2)	0.0489 (9)	
H2	0.509986	0.562449	0.408373	0.059*	
O3	0.3190 (4)	0.3762 (3)	0.48200 (17)	0.0596 (8)	
C3	0.6447 (5)	0.4554 (6)	0.3617 (3)	0.0581 (12)	
H3	0.726078	0.463818	0.397392	0.07*	
C4	0.6522 (5)	0.3836 (6)	0.3024 (3)	0.0647 (13)	
H4	0.738876	0.34243	0.298079	0.078*	
C7	0.2544 (4)	0.5741 (4)	0.3257 (2)	0.0442 (9)	
C6	0.4034 (5)	0.4366 (5)	0.2548 (2)	0.0496 (10)	
C5	0.5325 (5)	0.3725 (6)	0.2497 (3)	0.0613 (12)	
H5	0.537768	0.321576	0.21015	0.074*	
C8	0.2122 (4)	0.5470 (4)	0.3950 (2)	0.0407 (8)	
C9	0.0444 (4)	0.5578 (5)	0.3866 (3)	0.0528 (11)	
H9A	−0.000447	0.601873	0.342983	0.063*	
H9B	0.002167	0.464174	0.389089	0.063*	
C10	0.0274 (5)	0.6489 (5)	0.4456 (3)	0.0600 (12)	
H10	−0.060286	0.663186	0.458092	0.072*	
C11	0.1492 (6)	0.7068 (5)	0.4781 (3)	0.0612 (12)	
H11	0.156817	0.766726	0.515951	0.073*	
C12	0.2749 (5)	0.6666 (5)	0.4479 (2)	0.0506 (10)	
H12A	0.354422	0.630768	0.483133	0.061*	
H12B	0.307966	0.747473	0.425131	0.061*	
C13	0.2590 (4)	0.4002 (4)	0.4236 (2)	0.0397 (8)	
C14	0.2441 (5)	0.1481 (5)	0.3876 (3)	0.0530 (10)	
C15	0.1631 (9)	0.0987 (6)	0.4407 (4)	0.091 (2)	
H15A	0.070819	0.145176	0.432802	0.137*	
H15B	0.217624	0.122419	0.485973	0.137*	
H15C	0.149492	−0.002829	0.437233	0.137*	
C16	0.1791 (7)	0.0833 (6)	0.3183 (3)	0.0763 (16)	
H16A	0.079978	0.112254	0.304044	0.114*	
H16B	0.184221	−0.018889	0.321627	0.114*	
H16C	0.231621	0.115416	0.285077	0.114*	
C17	0.4050 (6)	0.1218 (6)	0.4074 (4)	0.0851 (19)	
H17A	0.450457	0.165224	0.373923	0.128*	
H17B	0.423246	0.020849	0.408884	0.128*	
H17C	0.443561	0.16255	0.451908	0.128*	

### Atomic displacement parameters (Å^2^)

**Table:**

	*U*^11^	*U*^22^	*U*^33^	*U*^12^	*U*^13^	*U*^23^
I1	0.0721 (3)	0.1182 (4)	0.0569 (2)	−0.0009 (2)	−0.00751 (17)	−0.0218 (2)
O1	0.0613 (19)	0.078 (2)	0.065 (2)	0.0208 (17)	0.0140 (17)	0.0253 (18)
C1	0.0403 (19)	0.045 (2)	0.042 (2)	−0.0023 (16)	0.0093 (16)	0.0044 (17)
O2	0.0465 (14)	0.0380 (14)	0.0462 (15)	−0.0051 (11)	0.0046 (12)	−0.0032 (12)
C2	0.041 (2)	0.059 (3)	0.046 (2)	−0.0017 (18)	0.0091 (17)	−0.002 (2)
O3	0.074 (2)	0.0489 (17)	0.0468 (18)	0.0025 (14)	−0.0058 (15)	0.0017 (14)
C3	0.036 (2)	0.079 (3)	0.058 (3)	−0.002 (2)	0.0082 (19)	0.004 (2)
C4	0.046 (2)	0.083 (4)	0.068 (3)	0.010 (2)	0.019 (2)	0.002 (3)
C7	0.040 (2)	0.046 (2)	0.045 (2)	−0.0018 (17)	0.0072 (17)	0.0028 (18)
C6	0.045 (2)	0.058 (3)	0.043 (2)	−0.0054 (18)	0.0062 (17)	−0.0026 (19)
C5	0.067 (3)	0.069 (3)	0.053 (3)	0.003 (2)	0.024 (2)	−0.010 (2)
C8	0.0350 (18)	0.043 (2)	0.045 (2)	−0.0019 (15)	0.0090 (15)	−0.0025 (17)
C9	0.0351 (19)	0.063 (3)	0.062 (3)	0.0045 (18)	0.0126 (18)	0.005 (2)
C10	0.059 (3)	0.061 (3)	0.066 (3)	0.020 (2)	0.028 (2)	0.014 (2)
C11	0.084 (3)	0.044 (2)	0.062 (3)	0.012 (2)	0.029 (3)	0.001 (2)
C12	0.057 (2)	0.043 (2)	0.053 (2)	−0.0029 (18)	0.015 (2)	−0.0033 (19)
C13	0.0361 (18)	0.042 (2)	0.041 (2)	−0.0002 (15)	0.0096 (16)	0.0013 (16)
C14	0.057 (2)	0.035 (2)	0.068 (3)	−0.0008 (18)	0.016 (2)	−0.004 (2)
C15	0.143 (6)	0.051 (3)	0.096 (5)	−0.019 (3)	0.061 (5)	0.000 (3)
C16	0.087 (4)	0.057 (3)	0.082 (4)	−0.007 (3)	0.013 (3)	−0.024 (3)
C17	0.067 (3)	0.063 (3)	0.117 (5)	0.021 (3)	0.002 (3)	−0.008 (3)

### Geometric parameters (Å, º)

**Table:**

I1—C6	2.096 (4)	C9—H9A	0.97
O1—C7	1.193 (5)	C9—H9B	0.97
C1—C2	1.382 (6)	C10—C11	1.312 (8)
C1—C6	1.395 (6)	C10—H10	0.93
C1—C7	1.514 (5)	C11—C12	1.497 (6)
O2—C13	1.331 (5)	C11—H11	0.93
O2—C14	1.476 (5)	C12—H12A	0.97
C2—C3	1.382 (6)	C12—H12B	0.97
C2—H2	0.93	C14—C16	1.508 (8)
O3—C13	1.197 (5)	C14—C15	1.511 (8)
C3—C4	1.373 (7)	C14—C17	1.512 (7)
C3—H3	0.93	C15—H15A	0.96
C4—C5	1.371 (7)	C15—H15B	0.96
C4—H4	0.93	C15—H15C	0.96
C7—C8	1.540 (6)	C16—H16A	0.96
C6—C5	1.389 (7)	C16—H16B	0.96
C5—H5	0.93	C16—H16C	0.96
C8—C13	1.516 (5)	C17—H17A	0.96
C8—C12	1.561 (6)	C17—H17B	0.96
C8—C9	1.568 (5)	C17—H17C	0.96
C9—C10	1.489 (7)		
			
C2—C1—C6	118.2 (4)	C10—C11—C12	113.1 (4)
C2—C1—C7	118.9 (4)	C10—C11—H11	123.4
C6—C1—C7	122.9 (4)	C12—C11—H11	123.4
C13—O2—C14	122.5 (3)	C11—C12—C8	103.4 (4)
C1—C2—C3	121.7 (4)	C11—C12—H12A	111.1
C1—C2—H2	119.2	C8—C12—H12A	111.1
C3—C2—H2	119.2	C11—C12—H12B	111.1
C4—C3—C2	119.4 (4)	C8—C12—H12B	111.1
C4—C3—H3	120.3	H12A—C12—H12B	109.1
C2—C3—H3	120.3	O3—C13—O2	125.3 (4)
C5—C4—C3	120.2 (4)	O3—C13—C8	124.9 (4)
C5—C4—H4	119.9	O2—C13—C8	109.7 (3)
C3—C4—H4	119.9	O2—C14—C16	102.5 (4)
O1—C7—C1	121.2 (4)	O2—C14—C15	109.0 (4)
O1—C7—C8	121.5 (4)	C16—C14—C15	110.4 (5)
C1—C7—C8	117.1 (3)	O2—C14—C17	109.1 (4)
C5—C6—C1	119.9 (4)	C16—C14—C17	111.1 (5)
C5—C6—I1	116.5 (3)	C15—C14—C17	114.1 (6)
C1—C6—I1	123.4 (3)	C14—C15—H15A	109.5
C4—C5—C6	120.5 (4)	C14—C15—H15B	109.5
C4—C5—H5	119.8	H15A—C15—H15B	109.5
C6—C5—H5	119.8	C14—C15—H15C	109.5
C13—C8—C7	111.9 (3)	H15A—C15—H15C	109.5
C13—C8—C12	111.1 (3)	H15B—C15—H15C	109.5
C7—C8—C12	110.5 (3)	C14—C16—H16A	109.5
C13—C8—C9	107.8 (3)	C14—C16—H16B	109.5
C7—C8—C9	110.7 (3)	H16A—C16—H16B	109.5
C12—C8—C9	104.7 (3)	C14—C16—H16C	109.5
C10—C9—C8	103.7 (4)	H16A—C16—H16C	109.5
C10—C9—H9A	111.0	H16B—C16—H16C	109.5
C8—C9—H9A	111.0	C14—C17—H17A	109.5
C10—C9—H9B	111.0	C14—C17—H17B	109.5
C8—C9—H9B	111.0	H17A—C17—H17B	109.5
H9A—C9—H9B	109.0	C14—C17—H17C	109.5
C11—C10—C9	112.8 (4)	H17A—C17—H17C	109.5
C11—C10—H10	123.6	H17B—C17—H17C	109.5
C9—C10—H10	123.6		
			
C6—C1—C2—C3	−1.1 (7)	C13—C8—C9—C10	104.1 (4)
C7—C1—C2—C3	−179.9 (4)	C7—C8—C9—C10	−133.3 (4)
C1—C2—C3—C4	−0.9 (7)	C12—C8—C9—C10	−14.3 (5)
C2—C3—C4—C5	0.6 (8)	C8—C9—C10—C11	9.5 (5)
C2—C1—C7—O1	130.7 (5)	C9—C10—C11—C12	−0.2 (6)
C6—C1—C7—O1	−48.0 (6)	C10—C11—C12—C8	−9.2 (5)
C2—C1—C7—C8	−45.7 (5)	C13—C8—C12—C11	−101.9 (4)
C6—C1—C7—C8	135.7 (4)	C7—C8—C12—C11	133.3 (4)
C2—C1—C6—C5	3.4 (6)	C9—C8—C12—C11	14.1 (4)
C7—C1—C6—C5	−177.9 (4)	C14—O2—C13—O3	−0.9 (6)
C2—C1—C6—I1	179.2 (3)	C14—O2—C13—C8	−178.2 (3)
C7—C1—C6—I1	−2.2 (6)	C7—C8—C13—O3	133.6 (4)
C3—C4—C5—C6	1.7 (8)	C12—C8—C13—O3	9.6 (5)
C1—C6—C5—C4	−3.7 (7)	C9—C8—C13—O3	−104.5 (5)
I1—C6—C5—C4	−179.8 (4)	C7—C8—C13—O2	−49.1 (4)
O1—C7—C8—C13	150.6 (4)	C12—C8—C13—O2	−173.1 (3)
C1—C7—C8—C13	−33.0 (5)	C9—C8—C13—O2	72.8 (4)
O1—C7—C8—C12	−85.0 (5)	C13—O2—C14—C16	−180.0 (4)
C1—C7—C8—C12	91.3 (4)	C13—O2—C14—C15	63.0 (6)
O1—C7—C8—C9	30.4 (6)	C13—O2—C14—C17	−62.2 (6)
C1—C7—C8—C9	−153.2 (4)		

### Hydrogen-bond geometry (Å, º)

**Table:**

*D*—H···*A*	*D*—H	H···*A*	*D*···*A*	*D*—H···*A*
C2—H2···O3^i^	0.93	2.48	3.219 (5)	136

Symmetry code: (i) −*x*+1, −*y*+1, −*z*+1.
